# Supplementary material for: From sequence to enzyme mechanism using multi-label machine learning
Source: BMC Bioinformatics. 2014 May 19;15:150. doi: 10.1186/1471-2105-15-150 (PMC4229970; doi:10.1186/1471-2105-15-150)
Supplement: Additional file 2 — Java code of ml2db. Additional file ml2db_code.tar.gz contains the Java source code to run the multi-label machine learning experiments and save the results to database. The code’s Javadoc is included. [file 1471-2105-15-150-S2.zip › additional file 2/ml2db/ml2db/doc/mulan/data/class-use/LabelClustering.html]

Uses of Interface mulan.data.LabelClustering


JavaScript is disabled on your browser.


- Overview
- Package
- Class
- Use
- Tree
- Deprecated
- Index
- Help

- Prev
- Next

- Frames
- No Frames

- All Classes

## Uses of Interface mulan.data.LabelClustering

- Packages that use LabelClustering

  | Package | Description |
  | --- | --- |
  |  |  |
  | --- | --- |
  | mulan.classifier.meta |  |
  | mulan.data |  |
- - ### Uses of LabelClustering in mulan.classifier.meta

    Constructors in mulan.classifier.meta with parameters of type LabelClustering

    Constructor and Description

    `SubsetLearner(LabelClustering clusteringMethod,
    MultiLabelLearner multiLabelLearner,
    weka.classifiers.Classifier singleLabelClassifier)`

    Initialize the SubsetLearner with a label clustering method, multilabel
    and single label learners.
  - ### Uses of LabelClustering in mulan.data

    Classes in mulan.data that implement LabelClustering

    | Modifier and Type | Class and Description |
    | --- | --- |
    |  |  |
    | --- | --- |
    | `class` | `GreedyLabelClustering` A class for clustering dependent label pairs into disjoint subsets. |

- Overview
- Package
- Class
- Use
- Tree
- Deprecated
- Index
- Help

- Prev
- Next

- Frames
- No Frames

- All Classes
